# Dual-resonance optical fiber lossy mode resonance refractive index sensor

**DOI:** 10.1371/journal.pone.0334249

**Published:** 2025-10-27

**Authors:** Xupeng Wu, Xiaoshuang Dai, Jian Wang, Haipeng Ren, Xu Wang

**Affiliations:** 1 Department of Urology, The Second Hospital of Tianjin Medical University, Tianjin, China; 2 Tianjin Institute of Urology, The Second Hospital of Tianjin Medical University, Tianjin, China; 3 Department of Urology, Jincheng People’s Hospital, Shanxi, China; 4 School of Precision Instrument and Opto-electronics Engineering, Tianjin University, Tianjin China; Universiti Brunei Darussalam, BRUNEI DARUSSALAM

## Abstract

In this paper, we propose a dual-channel surface refractive index sensor based on optical fiber lossy mode resonance (LMR). A multilayer transmission matrix model is constructed to simulate and analyze the optical fiber LMR effect based on two semiconductor oxides, SnO_2_ and TiO_2_. Through the rational design of structural parameters, we successfully realize the coexistence of mutually independent LMRs on a single fiber, thereby enabling dual-channel sensing. The dual-channel optical fiber LMR sensor was prepared by electrostatically assembling TiO_2_ nanofilms and SnO_2_ nanofilms on the sidewall of the optical fiber. The refractive index sensitivity of the dual-channel optical fiber LMR was evaluated via wavelength interrogation, achieving dual-channel refractive index sensing. The refractive index sensitivities are 538.422 nm/RIU and 615.647 nm/RIU in the range of 1.3350 ~ 1.3742. The dual-channel optical fiber LMR refractometer designed in this investigation is a promising platform for the simultaneous detection of multiple protein species and heavy metal ions in biochemical applications.

## Introduction

Optical fiber sensing technology has attracted significant attention in recent decades because of its inherent advantages, including cost-effective operation, high detection sensitivity, a miniaturized form factor, inherent flexibility, platform simplicity, and label-free detection capabilities [1–6]. These characteristics have driven its widespread adoption across diverse applications, from industrial monitoring to biomedical diagnostics. Recent advances have further expanded the scope of optical fiber biosensing, with notable applications in quantitative measurements of proteins [7–9], aptamer-based recognition systems [10–12], single-molecule detection [13–15], and other biochemical analytes [16–18]. Among these methods, lossy mode resonance (LMR) has emerged as a highly promising approach for label-free biochemical sensing [19]. LMR is achieved by high-refractive-index materials used as a new cladding for fibers, such as semiconductors, metal oxides, or high-refractive-index polymer films. When light propagates through the fiber, periodic coupling between the guided modes supported by the core and the lossy modes supported by the high-refractive-index cladding results in attenuation at specific wavelengths. Optical fiber LMR sensing technology can directly sense the refractive index change of the sensor surface caused by molecular interactions, such as protein binding or analyte adsorption. Compared with conventional techniques, which have the advantages of compact size, low cost, lack of labelling, high sensitivity, ease of miniaturization, multiparameter, real-time in situ detection, etc., the optical fiber LMR sensing platform combines these attributes to achieve exceptional performance.

Del Villar’s group successfully prepared an optical fiber LMR sensor, which proved useful for refractive index sensing and showed promising prospects for further development [20]. In subsequent years, we have witnessed a growing number of investigations employing optical fiber LMR sensing theory to achieve refractive index sensing and biochemical sensing [21–26]. Current research efforts are primarily concentrated on the fiber structure design (D-shaped fibers and tapered fibers) and the exploitation of the lossy mode support layer to enhance the detection performance [23,27,28]. For instance, Heidarnia et al. [27] evaporated an arsenic trisulfide (As_2_S_3_) absorptive thin-film layer on the surface of a low-OH etched optical fiber. The effect of LMR attenuation induced by evanescent-wave absorption on refractive index measurements in aqueous environments was investigated. Fuentes et al. [28] proposed an improved full width at half maximum (FWHM) method applied to LMR generated by a D-shaped fiber in a reflective structure. The bandwidth characteristics of LMR improved from 106 nm to 53 nm when a laser-cleaner method was used. Despite the significant advancements in optical fiber LMR sensors for quantitative biomarker detection, current designs predominantly concentrate on the fabrication of isolated sensing regions and neglect the complexity of cancer pathogenesis that arises from the synergistic interplay of multiple biomarkers. This single-marker detection paradigm inherently limits diagnostic accuracy and clinical utility, as cancer progression and prognosis are governed by multifactorial networks involving proteins, nucleic acids, metabolites, and extracellular vesicles rather than isolated molecular targets. Therefore, the urgent need for multichannel optical sensors arises from the increasing demand for the simultaneous, label-free, and quantitative detection of multiple biomarkers, which are critical for unravelling complex pathophysiological processes and enabling precision diagnostics in clinical oncology.

Herein, we present a dual-channel optical-fiber LMR refractive index sensor achieved with SnO_2_ and TiO_2_. Numerical analysis confirms the independent dual-channel sensing capability on the same fiber platform, with distinct resonance wavelengths assigned to each sensor channel. A dual-channel optical fiber LMR sensor was prepared by electrostatically assembling TiO_2_ nanofilms and SnO_2_ nanofilms on the sidewall of an optical fiber using layer-by-layer self-assembly. Refractive index sensing experiments verify its dual-channel sensing effect. The proposed dual-channel optical fiber LMR sensor enables the label-free, simultaneous detection of multiple cancer biomarkers on a single fiber without channel switching delays. Single-fiber integration maintains compact dimensions, avoiding the bulkiness of multifiber bundles and addressing the critical need for multiparametric diagnostics in precision oncology.

## Construction of the LMR sensing model

**Fig 1:**
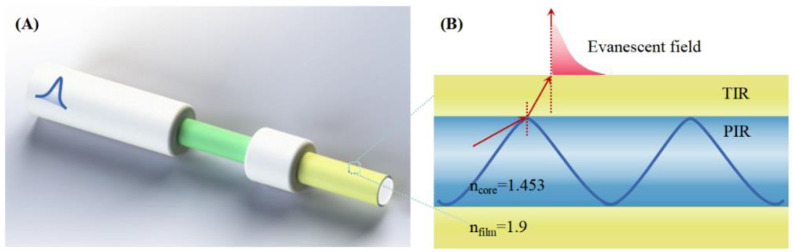
Basic principle of the optical fiber LMR sensor. **(A)** Schematic diagram of the dual-channel optical fiber LMR probe, where the two film colors represent TiO_2_ and SnO_2_. **(B)** General transmission principles of optical fiber LMR.

The basic principle of the optical-fiber LMR sensor is shown in Fig 1. The optical fiber LMR probe structure with an unclad core of the multimode fiber is depicted in Fig 1A. As the incident light travels into the unclad fiber region, the total internal reflection of the optical fiber is no longer satisfied since the high-refractive-index film acts as the new cladding layer. A certain portion of the light spills out of the fiber core and propagates in the high-refractive-index film, converting from guided modes to lossy modes. The interface between light propagating in a high-refractive-index film and the external environment satisfies the total internal reflection and generates an evanescent field, as shown in Fig 1B. The LMR effect occurs when the effective refractive index of the evanescent wave matches that of the lossy mode wave. This causes a redistribution of energy in the optical field. Sensing is achieved by wavelength interrogation, where the resonance wavelength of LMR varies with the effective refractive index of the thin film material, which depends highly on the refractive index of the film and the external analyte. As the parameters of the external analyte change, the resonance wavelength varies accordingly, resulting in the sensing capability. In general, the behaviour is characterized by a redshift of the resonance wavelength as the refractive index of the external analyte increases. Accordingly, changes in the refractive index of the analyte are recognized on the basis of the evolution of the resonance wavelength. In this study, to satisfy the need for independent dual sensing on the same fiber, two alternative versions of high-refractive-index films, TiO_2_ and SnO_2_, are designed to be deposited on the sidewall of the fiber, as illustrated in Fig 1A.

In this work, TiO_2_ and SnO_2_ thin films were used to realize optical fiber LMR sensors because of their advantageous properties: exceptional biocompatibility, high refractive index, and abundant surface defects that enhance analyte adsorption. These characteristics render them ideal candidates for versatile biological and chemical sensing applications. Critically, the deliberate selection of this material pair is fundamentally enabled by their stark contrast in complex permittivity. This difference ensures that their respective LMR resonances occur at distinct wavelengths within the spectrum, forming the foundation for achieving simultaneous, dual-channel operation on a single fiber platform. A key design consideration is then to fine-tune the film thicknesses for each material to optimize this inherent spectral separation.

**Fig 2:**
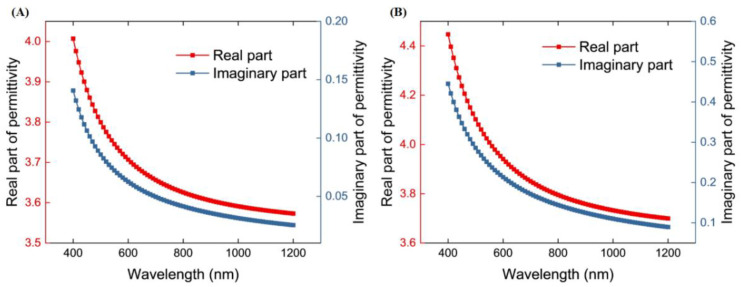
Dispersion curves for (A) SnO_2_ permittivity and (B) TiO_2_ permittivity in the visible to near-infrared region.

Different preparation processes are also important factors that affect the performance parameters of thin film materials. Here, the dispersion models developed in references [29,30] are chosen for the calculation of the complex permittivity, as presented in Fig 2. Analysis of the dispersion curves reveals that both materials exhibit LMR capabilities across the visible spectrum. Notably, the permittivity function of TiO_2_ has a larger real component than that of SnO_2_, directly correlating with its superior refractive index. In addition, the dispersion curves of both show a decreasing trend with increasing wavelength. The permittivity function is a key factor affecting the LMR resonance spectra. In this case, a larger real part corresponds to a higher refractive index. However, a larger imaginary part implies a wider FWHM. Therefore, such material-specific tuning strategies underscore the importance of tailoring LMR characteristics to match the demands of target applications, such as label-free biomarker detection in complex biological matrices.

To theoretically characterize the performance of the optical-fiber LMR sensor, we consider the Kretschmann configuration and determine the normalized spectral power at different wavelengths. The Sellmeier equation of the silica core is given by Zhao et al. [30]. The transmittance can be calculated numerically by the following integration:


$$T(\lambda )=\frac{\int_{\theta_{c}}^{90^{\circ }}p\left(\theta \right)R^{N\left(\theta \right)}\left(\theta ,\lambda \right)d\theta }{\int_{\theta_{c}}^{90^{\circ }}p\left(\theta \right)d\theta }$$ (1)

where θc=sin−1(ncl/ncln0\nulldelimiterspacen0) is the critical angle of total internal reflection of the fiber core layer and $n_{cl}$ and $n_{0}$ are the refractive indices of the fiber cladding and the core layer, respectively. $P\left(\theta \right)=\varepsilon_{0}\sin\theta \cos\theta /{\left(1-\varepsilon_{0}\cos^{2}\theta \right)}^{2}$ is the distribution function of the optical signal power on the fiber end facet, θ is the angle of incidence, and $\varepsilon_{0}$ is the dielectric constant of the fiber. $R^{N\left(\theta \right)}\left(\theta ,\lambda \right)=\left(R_{TM}^{N\left(\theta \right)}\left(\theta ,\lambda \right)+R_{TE}^{N\left(\theta \right)}\left(\theta ,\lambda \right)\right)/2$, which is a linear combination of the reflected optical power for the TE and the TM modes. The number of reflections can be expressed as N(θ)=L/(dtanθ), where L is the length of the sensing area of the LMR sensor and d is the diameter of the fiber.

In this N-layer reflection matrix model, the relation between the tangential components of the electric and magnetic fields at each boundary is shown in Eq. (2):


$$\left(\begin{array}{c} {}*20cU_{1}\\ V_{1} \end{array}\right)=M\left(\begin{array}{c} {}*20cU_{N-1}\\ V_{N-1} \end{array}\right)$$ (2)

where U and V are the tangential components of the electric and magnetic fields at the corresponding boundaries, respectively. M is the specific matrix of the N-layer mode, which is defined as follows:


$$M=\prod_{j=2}^{N-1}M_{j}=\prod_{j=2}^{N-1}\left(\begin{array}{cc} {}*20l\cos\beta_{j} & -\frac{i}{q_{j}}\sin\beta_{j}\\ -iq_{j}\sin\beta_{j} & \cos\beta_{j} \end{array}\right)=\left(\begin{array}{cc} {}*20cM_{11} & M_{12}\\ M_{21} & M_{22} \end{array}\right)$$ (3)

in which


$$\beta_{j}=\frac{2\pi }{\lambda }n_{j}\cos\theta (Z_{j}-Z_{j-1})=\frac{2\pi d_{j}}{\lambda }\sqrt{\varepsilon_{j}-n_{1}^{2}\;\sin^{2}\;\theta }$$ (4)

With TE-mode polarization,


$$q_{j}=\sqrt{\varepsilon_{j}-n_{1}^{2}\;\sin^{2}\;\theta }$$ (5)

With TM-mode polarization,


$$q_{j}=\sqrt{\frac{\mu_{j}}{\varepsilon_{j}}}\cos\theta =\frac{\sqrt{\varepsilon_{j}-n_{1}^{2}\;\sin^{2}\;\theta }}{\varepsilon_{j}}$$ (6)

The reflectance of the configuration can be calculated by the following equation:


$$R_{TE/TM}={\left|r_{TE/TM}\right|}^{2}={\left|\frac{\left(M_{11}+M_{12}q_{N})q_{1}-(M_{21}+M_{22}q_{N}\right)}{\left(M_{11}+M_{12}q_{N})q_{1}+(M_{21}+M_{22}q_{N}\right)}\right|}^{2}$$ (7)

According to the above model, the excitation conditions of LMR with TE-mode polarization and TM-mode polarization are different. The generation of resonance is affected mainly by multiple factors, such as the angle of incidence, layer thickness, dielectric constant, and refractive index. In fact, various lossy films are selected as needed to realize the LMR effect. The resonance wavelength can be modulated by changing the thickness of the loss layer and the refractive index of the sensing layer, making it possible to achieve the dual-channel sensor.

## Numerical simulations and analysis

### Effect of the film thickness

**Fig 3:**
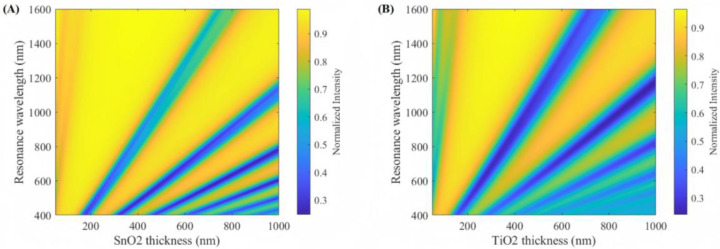
Evolution of the resonance wavelength of the optical fiber LMR sensor with film thickness. **(A)** SnO_2_ as the lossy layer. **(B)** TiO_2_ as the lossy layer.

To optimize the configuration of a noninterfering dual-channel optical fiber LMR sensor, it is essential to systematically analyze the thickness-dependent modulation of the resonance wavelength in optical fiber systems. In this study, SnO_2_ and TiO_2_ thin films are employed as lossy mode supporting layers, and their distinct complex permittivity characteristics are leveraged to explore their unique resonance behaviors. The evolution of the resonance wavelength of the LMR sensor with respect to the film thickness, which arises from the combined influence of TE-mode and TM-mode polarized light interactions, is shown in Fig 3. The resonance wavelength is the spectral position corresponding to the minimum normalized intensity in the output spectrum, as dictated by the destructive interference condition in the fiber core−cladding interface. The resonance wavelength is modulated by the film thickness. As the thickness of the film layer increases, the resonance wavelength of the first-order resonance progressively redshifts. This redshift is attributed to the enhanced optical energy dissipation in thicker films. In addition, higher-order resonances are excited. As a result, the distinct complex permittivity functions of SnO_2_ and TiO_2_ cause their resonance wavelengths to respond differently to thickness variations. This inherent difference enables the selection of appropriate thicknesses that position the respective resonances at spectrally distant wavelengths.

### Spectral characteristics of the optical fiber dual-channel LMR sensor

**Fig 4:**
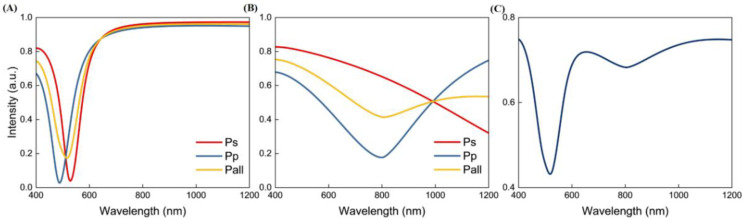
The optical fiber LMR spectra for polarization excitation in TE mode and TM mode. **(A)** LMR spectrum of the optical fiber at a SnO_2_ thickness of 235 nm. **(B)** LMR spectrum of the optical fiber at a TiO_2_ thickness of 75 nm. **(C)** LMR spectrum of the dual-channel sensor with the same fiber.

After determining the relationship between the resonance wavelength and film thickness, we selected a SnO_2_ thickness of 235 nm and a TiO_2_ thickness of 75 nm as the thickness of the lossy layer. The LMR spectra of the optical fiber for polarization excitation in TE mode and TM mode are shown in Fig 4, with all the resonance wavelengths corresponding to wavelengths of the visible band. In Fig 4A, although there is a difference between the dips in resonance wavelength for polarized light excitation in the TE mode and the TM mode, the resonance excitation when no polarized light is incident is not affected. The resonance wavelength of the SnO_2_-coated optical fiber sensor is 516 nm. The LMR spectrum of the TiO_2_-coated optical fiber with a resonance wavelength position of 806 nm is shown in Fig 4B. TE-mode polarized light fails to excite the LMR in the range of 400–1200 nm. The LMR of this structure is only excited by the TM-mode polarized light. As a result, its optical power is increased when no-polarized light is incident. A slight increase in the FWHM of the spectrum can be observed. The LMR spectrum of the dual-channel sensor with the same fiber is shown in Fig 4C, demonstrating its achievement of complete resonance wavelength separation. The significant spectral separation between the resonances stems from their fundamentally different material dispersion characteristics and optimized film thickness. As shown in Fig 4C, the spectral separation between the two resonances is 286 nm. The FWHM values of the dual resonances are 93 nm and 121 nm, respectively. The resonance spacing far exceeds the FWHM of the individual resonances, ensuring minimal spectral overlap and preventing resonance interference. This lays the foundation for the detection of multiple antigens by immobilizing these two antibodies in different sensing regions.

### Finite element analysis of LMR

**Fig 5:**
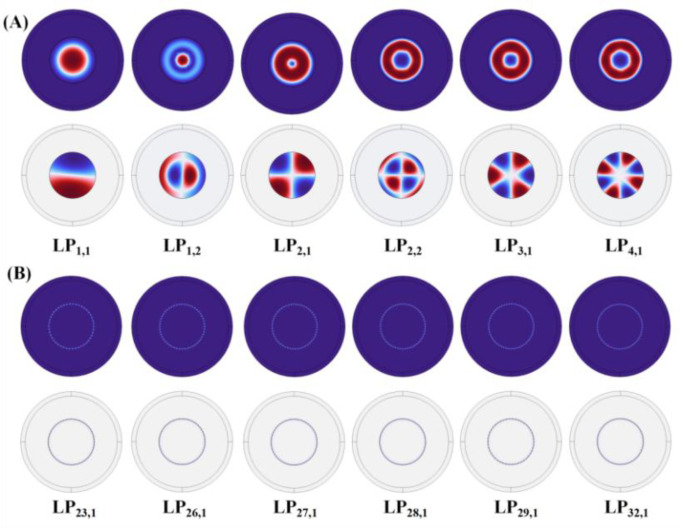
Series transverse electric field distribution and longitudinal component profiles of different LP modes. (A) guided modes. (B) lossy modes.

To obtain a more intuitive understanding of the intrinsic mechanism of the LMR effect in optical fibers, the specific absorption sensing mechanism was investigated via the finite element analysis (FEA) method. In the calculations, the LMR configuration of the optical fiber is sequentially divided into the following four layers: a 50 µm fiber core, a 130 nm lossy film, a surrounding dielectric layer with a thickness of 50 µm, and a perfect matching layer (PML) with a thickness of 10 µm. The complex permittivity of the SnO_2_ model based on the Lorentz model is used [29] in the calculation. The refractive index of the sensing region is set to 1.333. Finite element model analysis calculations are performed according to the set parameters. The transverse electric field distribution and longitudinal component profiles of the guided modes (LP_11_, LP_21_, LP_31_, LP_12_, and LP_22_) in the core region and the lossy modes (LP_44,1_, LP_40,1_, LP_36,1_, LP_35,1_, and LP_31,1_) in the SnO_2_-coated region are investigated in Fig 5. This analysis reveals energy transfer from the core modes to the lossy modes.

## Experimental results and discussion

### Sensor preparation and experimental system construction

In this experiment, the core/cladding diameter of the polymer clad fiber used to fabricate the reflective sensor is 400/430 µm. The TiO_2_/PSS composite film and SnO_2_/PSS composite film are coated on the bare core with different decladding regions. Unlike that of the transmissive probe, the process used to coat this reflective probe is simple, and a uniform coating can be easily formed by using the lift-off coating technique. Specifically, the present work focuses on the deposition of TiO_2_/PSS composite films and SnO_2_/PSS composite films on fiber cores using the electrostatic assembly method. This method overcomes the disadvantage of the conventional sputtering scheme, in which the sidewalls of cylindrical fibers are unevenly coated. In addition, electrostatic assembly works at room temperature, so it does not damage the fiber structure. For the deposition of the TiO_2_/PSS composite film, an aqueous PSS solution is used as the anionic solution, and a sonicated TiO_2_ nanodispersion is used as the cationic solution. The pH of the anionic and cationic solutions is adjusted to 2.0. First, the fiber core is immersed in 20 mg/mL TiO_2_ solution for 3 min, and the fiber is lifted using a lift coating machine at a speed of 1 mm/s. The fiber core is then rinsed with acidic deionized water for 1 min to remove unattached material from the surface and dried for 1 min. Afterwards, the core is immersed in 5 mg/mL PSS solution for 2 min, and the fiber is lifted again at a speed of 1 mm/s. The above cleaning and drying steps are repeated, and the deposition of the TiO_2_/PSS composite film can be completed. For the deposition of the SnO_2_/PSS composite film, a chitosan solution is used as a dispersant for the SnO_2_ powder. Chitosan is characterized by favourable film-forming properties, viscosity and a positive charge in acidic environments. First, 250 mg of chitosan powder is dissolved in 50 mL (4%) of acetic acid solution and stirred continuously for 30 min. After that, 500 mg of SnO_2_ powder is added to the chitosan solution and sonicated for 1 h to obtain a uniformly distributed SnO_2_ solution. The -NH_2_ on the molecular chain of chitosan is protonated to -NH^3^+ in an acidic environment. This positive functional group binds to negatively charged PSS through strong electrostatic interactions. The fiber is immersed in the ultrasonically dispersed SnO_2_/chitosan solution for 3 min, and the fiber is subsequently rinsed with acidic deionized water for 1 min to remove the unattached material from the surface of the fiber and dried for 1 min. The fiber is then immersed in 5 mg/mL PSS solution for 2 min. The above cleaning and drying steps are repeated, and the deposition of the SnO_2_/PSS composite film is completed.

**Fig 6:**
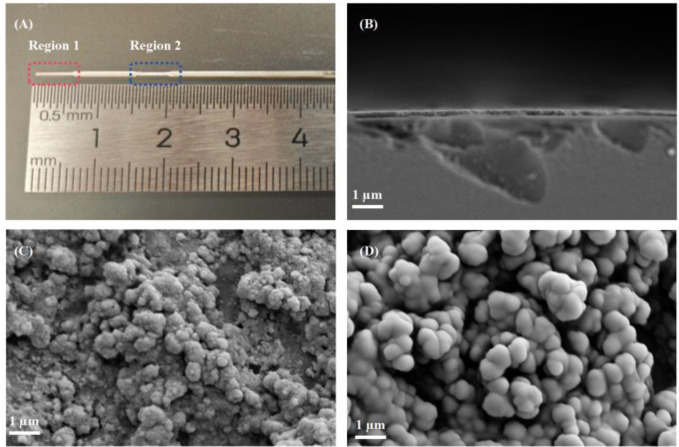
Characterization of the dual-channel optical-fiber LMR sensor. **(A)** Physical diagram of the dual-channel optical-fiber LMR sensor. **(B)** SEM image of the sensor end face. **(C)** TiO_2_ nanoparticles on the sidewall of the sensor. **(D)** SnO_2_ nanoparticles on the sidewall of the sensor.

A physical diagram of the dual-channel optical fiber LMR sensor is shown in Fig 6A. The circles represent the two sensing regions where LMR is realized with TiO_2_ and SnO_2_. The sensor end faces and sidewalls are characterized by scanning electron microscopy (SEM). Fig 6B clearly reveals a well-defined boundary between the fiber core and the coating layer, confirming the precise deposition of the films. Moreover, the film exhibits a uniform and continuous morphology on the fiber sidewall. In Fig 6C and 6D, the nanostructures of both materials are clearly resolved, with particle sizes averaging approximately 0.4 μm following ultrasonic dispersion, which contributes to the consistent surface roughness observed. Therefore, the SEM images confirm the successful preparation of the dual-channel optical fiber LMR sensor. Critically, no visible cracks, pinholes, or delamination are present, indicating excellent adhesion and structural integrity of the nanofilms. This morphological uniformity is essential for achieving reproducible LMR, as it minimizes spurious light scattering and ensures a consistent evanescent field interaction at the coating-analyte interface. The SEM analysis thus conclusively verifies the successful fabrication and robust quality of the dual-channel sensor.

**Fig 7:**
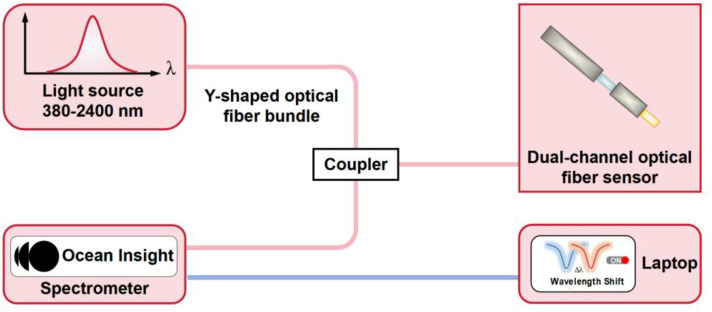
Schematic diagram of the dual-channel optical fiber LMR sensing experimental setup.

The refractive index sensing experimental setup is shown in Fig 7. The light emitted from the HL-2000 halogen light source is transmitted to the reflective dual-channel optical fiber LMR sensor through the Y-type fiber bundle with a matched core diameter and then reflected by the Y-type fiber bundle to the microspectrometer. Data acquisition of the resonance wavelength is performed by the upper computer software. The dual-channel optical fiber LMR sensor is placed in glycerol solutions of different mass concentrations for refractive index sensing experiments, and the refractive index is calibrated by an Abbe refractometer. Notably, before the next refractive index measurement, the sensor is washed with deionized water for 1 min and dried for 30 s. This operation is repeated three times to ensure the accuracy of the sensing results.

### Sensing performance of the optical-fiber dual-channel LMR sensor

**Fig 8:**
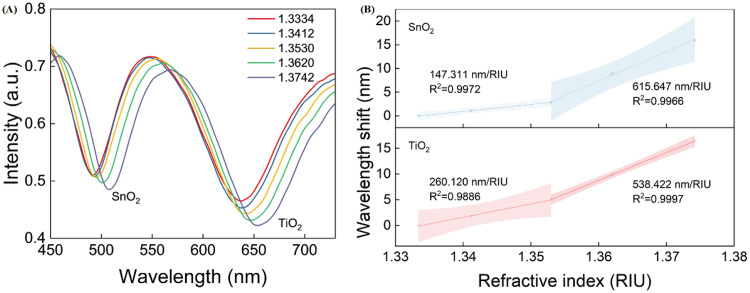
Refractive index sensing performance of the optical-fiber dual-channel LMR sensor. **(A)** Measured normalized spectra of the dual-channel sensor with the same fiber in samples with different RIs. **(B)** Calibration curves of resonance wavelength shifts and RIs.

**Fig 9:**
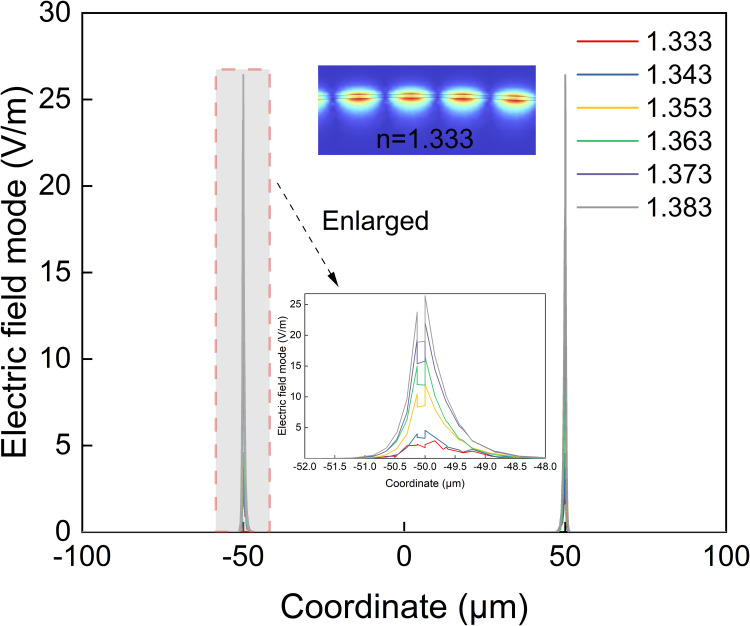
Simulated electric field mode across the sensor under different refractive indices. **Inset: enlarged line plot (bottom) and detailed field map at n = 1.333 (top)**.

In the refractive index sensing experiment, to construct a detection system with gradient refractive index distribution, glycerol (refractive index of ~1.474) and deionized water (refractive index of ~1.333) are mixed at different ratios. A mixed solution of glycerol and deionized water at different concentrations can be prepared by a magnetic stirrer, and the refractive index can be calibrated by an Abbe refractometer in the range of 1.3334 ~ 1.3742. The normalized spectrum of the optical fiber dual-channel sensor is measured by placing it in solutions with different refractive indices at a constant temperature. As shown in Fig 8A, the dual-channel sensing effect is realized by SnO_2_ and TiO_2_ as resonance excitation films. At the refractive index of 1.333, the resonance spectrum exhibits excellent spectral performance, with a full width at half maximum (FWHM) values of 45.486 nm for the SnO_2_ channel and 72.641 nm for the TiO_2_ channel. When the refractive index increases from 1.3334 to 1.3742, the resonance wavelengths of both channels clearly redshift, which is attributed to the increase in the effective refractive index of the evanescent field due to the increase in the refractive index of the medium. Furthermore, the wavelength required to satisfy the phase-matching condition of LMR is shifted towards the longwave direction. The resonance wavelength is extracted to obtain its shifts at different refractive indices, as shown in Fig 8B. For each refractive index point in the calibration range, we conduct thirty independent measurements. The shaded portions of the figure represent 95% confidence intervals. The refractive index sensitivity can be obtained by calculating the slope through segmental fitting. The refractive index sensitivities of different sensing channels significantly differ because of the differences in the complex dielectric functions of semiconductor materials. The results show that in the range of 1.3334 ~ 1.3530, the refractive index sensitivity achieved by TiO_2_ as the resonance excitation layer is 260.120 nm/RIU (R^2^ = 0.9886). The maximum standard deviation is 0.25 nm. Moreover, the refractive index sensitivity achieved by using SnO_2_ as the resonance excitation layer is 147.311 nm/RIU (R^2^ = 0.9972). The maximum standard deviation is 0.22 nm. The sensitivities of dual-channel sensing are substantially improved in the range of 1.3350 ~ 1.3742. This enhancement can be attributed to the reduced refractive index contrast between the fiber core and the external medium. As this contrast diminishes, optical confinement weakens, allowing the evanescent field to extend further into the external environment, as shown in Fig 9. This leads to a larger overlap integral between the electromagnetic field and the analyte, significantly enhancing the sensitivity of the sensor to changes in the external refractive index. In the range of 1.3350 ~ 1.3742, the refractive index sensitivity achieved by using TiO_2_ as the resonance excitation layer is 538.422 nm/RIU (R^2^ = 0.9966). The maximum standard deviation is 0.25 nm. Moreover, the refractive index sensitivity achieved by using SnO_2_ as the resonance excitation layer is 615.647 nm/RIU (R^2^ = 0.9997). The maximum standard deviation is 0.23 nm. The results of the refractive index response experiments verify that the optical fiber dual-channel LMR sensor is promising for applications in dual-parameter detection.

Previous studies on refractive index sensing using optical fiber LMR are listed in Table 1. Researchers tend to achieve highly sensitive sensing through techniques such as structure design and material sensitization. However, the advantages of multichannel sensing have been neglected in related research. The proposed dual-channel optical fiber LMR sensor in this study has excellent comprehensive performance. It is promising for application to the label-free simultaneous detection of multiple cancer biomarkers on a single fiber without channel switching delay. In future studies, we will introduce tapered optical fibers to improve the light‒substance interaction ability and thus enhance the detection sensitivity. The proposed dual-channel sensor is expected to provide a reliable solution for biomedical detection.

**Table 1:** Comparison of the proposed dual-channel sensor and other optical-fiber LMR sensors for refractive index sensing.

Scheme	Number of channels	Refractive index range	Sensitivity (nm/RIU)
Tapered optical fiber LMR sensor coated with a V_2_O_5_ film [31]	1	1.3310 ~ 1.4290	2050
Plastic clad silica fiber LMR sensor coated with a ZnO film [32]	1	1.3330 ~ 1.4330	760
Double cladding fiber without a high-refractive index thin film [33]	1	1.3300 ~ 1.3900	1700
Dual-resonance optical fiber LMR probe [21]	2	1.3333 ~ 1.3380	206.657 (TM resonance wavelength)
			154.419 (TE resonance wavelength)
Dual-channel optical fiber LMR sensor (This work)	2	1.3334 ~ 1.3530	260.120 (TiO_2_ as the resonance excitation layer)
			147.311 (SnO_2_ as the resonance excitation layer)
		1.3350 ~ 1.3742	538.422 (TiO_2_ as the resonance excitation layer)
			615.647 (SnO_2_ as the resonance excitation layer)

## Conclusions

In conclusion, we introduced a dual-channel optical fiber LMR sensor scheme that can be used for immunoassays of multiple biomarkers of cancer. According to the multilayer transmission matrix model and the FEA model, the optical fiber LMR effect based on two semiconductor oxides, SnO_2_ and TiO_2_, was analysed. The optimally configured spectral characteristics of the optical fiber LMR from the dispersion model and coating material thickness improve the dual-channel sensing performance. A dual-channel optical fiber LMR sensor was prepared using layer-by-layer self-assembly, and its refractive index sensing performance was also verified. Furthermore, various specific antibodies were immobilized at different sensing regions of the same fiber to achieve the detection of multiple markers by the wavelength interrogation method. In turn, the actual antigen concentration in the serum was demodulated. The dual-channel optical-fiber LMR refractometer developed in this investigation is promising for liquid-phase dual-parameter detection, such as dual-protein detection and simultaneous temperature and refractive index detection.

## Supporting information

S1 File Data. (XLSX)
